# Examining Vaccine Hesitancy in Sub-Saharan Africa: A Survey of the Knowledge and Attitudes among Adults to Receive COVID-19 Vaccines in Ghana

**DOI:** 10.3390/vaccines9080814

**Published:** 2021-07-22

**Authors:** Theophilus Acheampong, Eli A. Akorsikumah, John Osae-Kwapong, Musah Khalid, Alfred Appiah, John H. Amuasi

**Affiliations:** 1 iRIS Research Consortium, 6 Ashur Suites, North Legon, Accra, Ghana; josaekwapong@gmail.com (J.O.-K.); khalid.musah@live.com (M.K.); alfredappiah17@gmail.com (A.A.); 2 Department of Agricultural and Food Economics, University for Development Studies, Nyankpala-Tamale P.O. Box 1350, Ghana; akorsikumah@gmail.com; 3 Department of Global Health, School of Public Health, Kwame Nkrumah University of Science and Technology (KNUST), Kumasi 00233, Ghana; amuasi@kccr.de; 4 Kumasi Center for Collaborative Research in Tropical Medicine (KCCR), Kumasi, Ghana; 5 Bernhard Nocht Institute of Tropical Medicine, Bernhard-Nocht-Straße 74, 20359 Hamburg, Germany

**Keywords:** COVID-19, public health, vaccine hesitancy, willingness, attitude, Ghana, Sub-Saharan Africa

## Abstract

The impact of COVID-19 vaccination programmes on disease transmission, morbidity and mortality relies heavily on the population’s willingness to accept the vaccine. We explore Ghanaian adult citizens’ vaccine hesitancy attitudes and identify the likelihood of participation or non-participation in the government’s effort to get citizens vaccinated. A fully anonymised cross-sectional online survey of 2345 adult Ghanaians was conducted from 23 to 28 February 2021. Differences in intentions regarding COVID-19 vaccination were explored using Pearson Chi-square tests. Additionally, multinomial logistic regression was used to analyse the factors associated with willingness to receive vaccines. Responses were weighted using the iterative proportional fitting technique to generate a representative sample. About half (51%) of mostly urban adult Ghanaians over 15 years are likely to take the COVID-19 vaccine if made generally available. Almost a fifth (21%) of the respondents were unlikely to take the vaccine, while another 28% were undecided. Additionally, we find differences in vaccine hesitancy among some socio-demographic characteristics such as age, gender, and primary sources of information. Attaining the proverbial 63% to 70% herd immunity threshold in Ghana is only possible if the preventive vaccination programmes are combined with an enhanced and coordinated public education campaign. Such a campaign should focus on promoting the individual and population-level benefits of vaccination and pre-emptive efforts towards addressing misinformation about vaccines.

## 1. Introduction

Coronavirus disease (COVID-19), a novel infectious disease, was declared a global pandemic on 11 March 2020 by the World Health Organisation [1]. On 12 March 2020, Ghana reported its first case of COVID-19, and since then, there have been 92,856 total confirmed cases with 90,480 recoveries and 783 deaths as of 3 May 2021 [2]. In response to the outbreak, several nation-states imposed various disease prevention and control measures such as restrictions on movement, including lockdowns [3,4,5]. While the impact of these measures varied between various countries, they were largely successful in reducing the rate of spread of the virus [6,7,8,9,10]. Authorities in Ghana closed land, air, and sea borders in mid-March 2021. Some weeks later, all educational institutions, including universities, high schools, and primary schools (public and private), were closed. Bans were also imposed on public gatherings. Some of these interventions were subsequently lifted on 20 April 2020 following a three-week restriction on movement within parts of the Greater Accra and Greater Kumasi metropolis—the two most populous regions in the country [11,12,13]. These public health measures were also imposed as a stop-gap measure against an anticipated development of safe and efficacious COVID-19 vaccines, without which containing the spread of the virus would be almost impossible.

Vaccination is often considered one of the most efficient means of preventing disease and is often a cost-effective tool for improving health at the population level [14,15,16]. Population-level immunity against infectious diseases is important because the likelihood of the spread of the disease in the population is reduced, as many potential hosts for the pathogen that causes the disease are immune [17]. This population-level immunity to an infectious disease (commonly known as herd immunity) can occur either through vaccination or natural immunity developed from prior infection [18]. The WHO recommends achieving herd immunity through vaccination instead of exposing the population to an infectious disease, as the latter runs the risk of causing unnecessary infections and deaths [18]. In order to achieve herd immunity through vaccination, a substantial proportion of the population needs to be vaccinated before infection rates start declining [18].

Nevertheless, the percentage of the population that needs to be immune to achieve herd immunity varies with each disease—usually 50% to 90% [18,19]. For example, herd immunity for polio and measles is reported at 80% and 95% of the population to be vaccinated, respectively [18]. For COVID-19, herd immunity thresholds are reported to be in the 60% to 70% range to break the transmission chain [20,21,22].

However, the success of COVID-19 vaccination programmes on disease transmission, morbidity, and mortality rely heavily on the population’s willingness to accept the vaccine. Some segments of the population may delay accepting a safe vaccine or refuse to be vaccinated despite the availability of vaccination services [23]. This is commonly known as vaccine hesitancy. Reasons provided for vaccine hesitancy include concerns or fears over the safety of vaccines, doubts or scepticism about the benefits of vaccines, religious and moral convictions, social norms and social pressure, among others [24,25,26,27]. This fear is also often expressed among persons who are vaccinated [28]. Generally, global vaccine hesitancy is often further fuelled by conspiracy theories, especially through social media channels [29,30,31,32,33]. Vaccine hesitancy is a global public health concern, especially because it has a strong potential to lead to vaccine refusal [33,34,35,36,37].

Vaccine hesitancy trends vary across the globe. A systematic review of vaccine acceptance rates found general COVID-19 acceptance rates greater than 70% among adult populations [32]. At the country level, the highest COVID-19 vaccine acceptance rates among adults were in Ecuador (97.0%), Malaysia (94.3%), Indonesia (93.3%), and China (91.3%) [32]. In comparison, the lowest vaccine acceptance rates were found in Kuwait (23.6%), Jordan (28.4%), Italy (53.7%), Russia (54.9%), Poland (56.3%), USA (56.9%), and France (58.9%) [32].

Additionally, Chen et al. [38] found that 83.8% of 3195 Chinese adults were found to be willing to receive a COVID-19 vaccine, while another 76.6% believed having the vac-cine would benefit their health [38]. Nevertheless, another 74.9% were worried and or neutral in their views regarding the vaccine’s potential adverse effects [38].

Likewise, Khubchandani et al. [39] assessed COVID-19 vaccination hesitancy in the USA and found out that 52% of 1878 participants were very likely and another 27% somewhat likely to accept the vaccines. However, multiple regression analyses found heterogeneity in vaccine hesitancy by sex, education, employment, income, and other variables. In Sub-Saharan Africa, 52% of South Africans indicated that they would not take the COVID-19 vaccine [40]. Some of the major factors associated with vaccine hesitancy among South Africans were religious beliefs and fear of vaccination being a means for government tracking or surveillance. Furthermore, 50% of Zimbabweans indicated they would accept the vaccine, with 30% and 20% being unsure and rejecting the vaccine outright, respectively [40]. There are also varying acceptance levels, even among healthcare workers, whom ex-ante, one would have expected would be most willing to accept COVID-19 vaccines. For example, Gagneux-Brunon et al. [41] found a 75% intention to get vaccinated against COVID-19 among healthcare workers, but with significant variations between occupational categories. However, in a developing country context, Agyekum et al. [42] found that 39% of 234 healthcare workers intended to receive the COVID-19 vaccines, with differences in gender and healthcare category, and expressed concerns about the safety and side effects of the vaccines.

The government of Ghana announced in February 2021 that it was seeking to procure 17.6 million vaccine doses by the end of June 2021 [43]. The Ministry of Health further announced on 26 March 2021 that it expected to procure a total of 42 million COVID-19 vaccine doses [44]. This would be enough to inoculate about 63% of Ghana’s adult population above 15 years, allowing for the attainment of 60% to 70% herd immunity threshold, even without considering the proportion of the population with naturally acquired immunity. Ghana’s initial vaccine doses are being provided through COVAX and other bilateral sources such as Russia’s Sputnik V vaccine [45]. On 24 February 2021, Ghana became the first low-resource country outside India to receive COVID-19 vaccines through the COVAX facility, with mass vaccination beginning on 1 March 2021 [46,47]. The country initially received 600,000 doses of the AstraZeneca/Oxford vaccine licensed to the Serum Institute of India [47]. The country received another 350,000 doses of the AstraZeneca coronavirus vaccine on 7 May 2021, initially deployed to the Democratic Republic of Congo but could not be utilised due to logistical challenges [43,48]. According to Ghana Health Service data, as of 30 April 2021, Ghana had administered 849,527 first doses of the vaccines, comprising those sourced via COVAX and others procured directly or received as donations, including Russia’s Sputnik V vaccine. Available reports indicate that over 60% of Ghana’s first phase target population and 90% of all health workers had received first doses of the vaccine as of 27 April 2021 [49].

Despite the positive initial rollout, concerns remain regarding vaccine hesitancy among Africa’s wider population, including Ghana, which could hinder an effective vaccine rollout [50,51]. We, therefore, conducted this survey to examine Ghanaians’ vaccine hesitancy attitudes and identify the likelihood of participation or non-participation as part of efforts to vaccinate citizens against COVID-19. Another distinguishing feature of this paper is that, unlike other works which only assess vaccine hesitancy on a binary scale using logistic regression, we trichotomise our key variable as likely (very likely or somewhat likely), mixed (undecided), or negative (somewhat unlikely or very unlikely). This allows a much more nuanced interpretation of vaccine hesitancy and implications for mitigation strategies and policies, especially among those who expressed mixed views (undecided) and those with negative (somewhat unlikely or very unlikely) views.

## 2. Materials and Methods

### 2.1. Study Design

A cross-sectional online survey was conducted over five days between 23 and 28 February 2021. The questionnaire was developed using Microsoft Forms and distributed through various media channels, including Facebook, WhatsApp, Twitter, LinkedIn, and email. Participation in this study was voluntary, and there was no compensation provided. In addition, all responses were fully anonymised. Our sampling is premised on the snowballing or chain-referral approach using virtual networks to reach the population with an online presence, allowing us to save time and cost [52]. While selection bias remains a threat to the representativeness of our survey, we argue that reaching out to Ghana’s online population is a beneficial exercise, as an indication of strong vaccine hesitancy within the “literate” population has serious consequences for negatively influencing the rest of the population [53,54,55]. Research also shows that misinformation or “fake news” tends to be shared or forwarded on such online social networks; thus, it is of public health value to collect or sample views of the population from such networks [56,57,58,59]. An additional important justification is that those who often access online sources of information are the most directly connected to networks outside of their immediate geographic location (especially outside the country) and are more likely to be influenced by online vaccine conspiracies emanating from far and wide. This also means that due to knowledge and power dynamics, some of the adult population who are not connected to the internet may be persuaded by those on social media via word of mouth to either take or not take the vaccine.

### 2.2. Study Setting and Sampling

Ghana is bounded on the West by Côte d’Ivoire, North by Burkina Faso, East by Togo, and the South by the Gulf of Guinea. Ghana is classified as a lower-middle-income country (LMIC) with a GNI per capita of $2220 as of 2019 [60]. The country has 16 administrative regions, with an estimated population of 30.96 million as of 2020 [61]. The survey instrument was designed and administered in English, Ghana’s official language. The anonymous questionnaire covered two parts: (1) attitudes and knowledge about COVID-19; and (2) information on respondents’ socio-demographic characteristics. The former includes the primary question of interest: that is, “how likely are you to take the COVID-19 vaccine if it was made generally available by the Government of Ghana?”. Responses were on a five-point Likert scale comprising, “very likely, somewhat likely, not decided, somewhat unlikely, and very unlikely”. Additional questions asked included: (1) reasons for wanting to take, being undecided or not wanting to take the COVID-19 vaccine (top 3 choices); (2) heard about the COVID-19 pandemic (Yes/No responses); (2) believe COVID-19 is real (Yes/No/Do not know responses); (3) tested for COVID-19 in the past one year, how many times tested and outcome of test; (4) primary sources for acquiring information or knowledge (4 choices). Gender, age, the highest educational attainment level, and residence region were part of the information collected to account for socio-demographics.

The questionnaire was developed guided by similar studies [39,40,62,63,64,65] and was reviewed by public health experts with several years’ experience developing and conducting such surveys. The questionnaire was also piloted with 20 persons before rollout—these participants were excluded from the survey. The initial minimum sample size of participants was calculated by the standard Cochran formula [66,67,68] given as follows:

(1) $$n_{o}=\frac{Z^{2}pq}{e^{2}},$$

where e = is the desired precision level (margin of error), p is the proportion of the population, q is 1−p, Z is the *z*-value as found in a *Z* table.

For our survey, a total of 2345 respondents filled out the close-ended questionnaire. This translates to a margin of error of ±2 percentage points at a 95% confidence level.

### 2.3. Statistical Analysis

We used descriptive statistical methods to summarise the survey data and describe the study participants’ demographic characteristics. This was followed by conducting chi-square tests to evaluate the associations between the socio-demographic characteristics and participants’ willingness to receive a COVID-19 vaccine, as documented in several studies [38,39,62]. Responses were compared for various socio-demographic characteristics by trichotomising the variable as likely (very likely or somewhat likely), mixed (not decided), or negative (somewhat unlikely or very unlikely) towards taking a COVID-19 vaccine, as indicated by the category of vaccine hesitancy. We subsequently employed multivariate logistic regression to identify the possible factors affecting vaccine hesitancy attitudes, with an alpha level set at 5% and *p*-value < 0.05 being considered statistically significant. The multinomial logit model specification is described in detail in Section 2.4. We weighted the survey responses using Ghana’s regional population breakdown of those aged 15 years and above and gender, based on publicly available data from the Ghana Statistical Service [61]. Weighting was based on the widely used iterative proportional fitting or raking technique for surveys and implemented with the “ipfraking” command in STATA 16 software package [69,70].

### 2.4. Multinomial Logit Model Specification

For all categories of vaccine hesitancy (Likely (1), Undecided (2), and Unlikely (3)), there is no natural ordering, but these choices provide the individual with the maximum utility. These options also reveal the strength of an individual’s preference of choosing a particular outcome [39]. The study employed the multinomial logit to analyse the determinants of likelihood to take the COVID-19 vaccine. For an individual i faced with j choices, the maximum utility for choosing j can be specified as:

(2) $$U_{ij}=Z_{ij}^{!}+\varepsilon_{ij}\;\;where\;Prob\left(U_{ij}>U_{ik}\right),k\neq j.$$

Following Greene [71], the multinomial logit can be specified as:

(3) $$(Y_{i}=j|x_{i})=\frac{exp\left(x_{i}^{!}\alpha_{j}\right)}{\sum_{j=1}^{3}exp\left(x_{i}^{!}\alpha_{j}\right)},\;j=1,2,3,$$

where *Y* denotes the probability of choosing from j choices with x as the set of individual characteristics and α econometric parameters. To ensure that Equation (3) is identified and further remove the indeterminacy, we set $\alpha_{k}$ = 0. Therefore, the probabilities can be determined as

(4) $$(Y_{i}=j|x_{i})=P_{ij}=\frac{exp\left(x_{i}^{!}\alpha_{j}\right)}{1+\sum_{j=2}^{3}exp\left(x_{i}^{!}\alpha_{k}\right)},\;\;j=1,2,3.$$

Equation (4) measures the change relative to the base. Again, the log-odds of the binomial with respect to alternative j relative to the base k can be computed in Equation (5).

(5) $$\ln\left[\frac{P_{ij}}{P_{ik}}\right]=x_{i}^{!}\left(\alpha_{j}-\alpha_{k}\right)=x_{i}^{!}\alpha_{j,\;}\mathrm{if}\;k=0.$$

Using the general log-likelihood of the binomial logit model, with an individual, i, choosing alternative j ($d_{ij}=1$ and 0 otherwise) gives:

(6) $$\ln L=\sum_{i=1}^{n}\sum_{j=0}^{J}d_{ij\;}\;\ln Prob(Y_{i}=j|x_{i}).$$

The derivative of from the log-likelihood is specified as

(7) $$\frac{\ln L}{\alpha_{j}}=\sum_{i=1}^{n}\left(d_{ij}-P_{ij}\right)w_{i}\;\mathrm{for}\;j=1,2,3.$$

## 3. Results

### 3.1. Participant Characteristics

A summary breakdown of the weighted demographic profiles for the 2345 survey respondents, which is consistent with Ghana’s population structure as of May 2020 [61], is as follows: Greater Accra had 19.77% of the respondents (down from 64.48% in unweighted sample); Ashanti 19.28% (up from 9.34% in unweighted sample), Eastern 10.72% (up from 4.86% in unweighted sample), Central 8.5% (up from 3.88% in unweighted sample), Western 6.89% (up from 3.45% in unweighted sample), Volta 6.43% (up from 2.81% in unweighted sample), and Northern 5.33% (up from 2.52% in unweighted sample), among others (Table 1). In terms of the age, 7.54% of the weighted sample were between 15 and 25 years, followed by 26–35 years (47.08%), 36–45 years (23.27%), 46–55 years (8.89%), and more than 55 years (13.22%)—Table 1. Regarding gender, the weighted proportion of male and female participants in this survey was 52.15% to 47.85%. On education, junior high school graduates represented 1.25% of the weighted sample, followed by respondents with secondary education (4.40%), tertiary (57.10%), postgraduate (36.34%), and other (0.91%).

**Figure 1:**
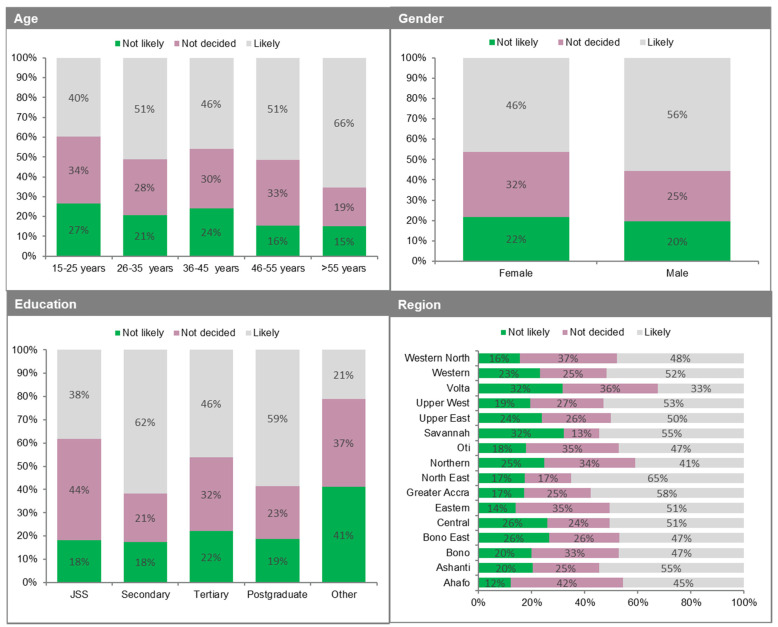
Likelihood of vaccine acceptance by demographics.

The results indicate that about half (5 in 10 or 51%) of mostly urban adult Ghanaians over 15 years are likely to take the COVID-19 vaccine if generally made available by the Government of Ghana (Table 1). This comprises those who answered “very likely or somewhat likely” to the question “how likely are you to take the COVID-19 vaccine if it was made generally available by the Government of Ghana?”. Another 28% or 3 in 10 of the sampled population indicated that they are “undecided” about taking the vaccine (Figure 1; Table 1). In comparison, another 21% (2 in 10 of the population) indicated that they are “somewhat unlikely or very unlikely” to take the vaccine (Table 1).

### 3.2. Vaccination Willingness and Hesitancy

Regarding the likelihood of taking the vaccine by the different demographic groups, we find some notable differences by gender, age, educational attainment, and region of residence. For example, on gender, there is an almost 9% difference between males (56%) and females (46%) on “very likely or somewhat likely” (Figure 1). On age, those above 55 years are the most “very likely or somewhat likely” to take the vaccine (66%) followed by the 46–55 year group (51%), 26–35 year group (51%), 36–45 year group (46%), and lastly the 15–25 year group (40%) (Figure 1). This observation is consistent with the literature where older population groups have been identified as relatively more at risk of requiring hospitalisation or dying if they contract COVID-19 [72]. Hence, it is not surprising that they are the most likely to take the vaccine. Such a finding is also consistent with, for example, the UK government’s strategy of vaccinating most of the vulnerable population first, including older persons and those with underlying health conditions [73]. Regarding the educational level, we find that respondents with a high school (secondary) degree are the most likely to take the vaccine at 62% (Figure 1). This is followed by postgraduate (master’s) degree (59%), tertiary (bachelor’s) degree (51%), junior secondary/high school (JSS) and below (38%), and lastly, other degrees (21%). Again, we posit that increased educational attainment levels are likely to drive vaccine uptake, all things being equal.

Lastly, as also shown in Figure 1, the top five regions most likely to take the COVID-19 vaccine are North East (65.10%), Greater Accra (57.87%), Ashanti (54.67%), Savannah (54.55%), and Upper West (53.08%). The bottom five regions were Bono (47.16%), Bono East (47.02%), Ahafo (45.41%), Northern (41.09%), and Volta (32.50%). From Figure 3, the results of the test of the relationship between the confirmed case rate in regions (that is, the cumulative case counts per 100,000 people) and the share of their population willing to be vaccinated were mixed. Whereas regions such as Greater Accra, Ashanti and Western who had the highest case rates had a higher share of the population willing to be vaccinated, others with lower case rates such as North East and Savannah also had a higher population share willing to be vaccinated. This is further corroborated in Table 2, which shows that differences between regions are not statistically significant—in fact, responses are fairly similar across regions. These factors are all empirically tested in the next Section 3.3 and discussed.

**Figure 2:**
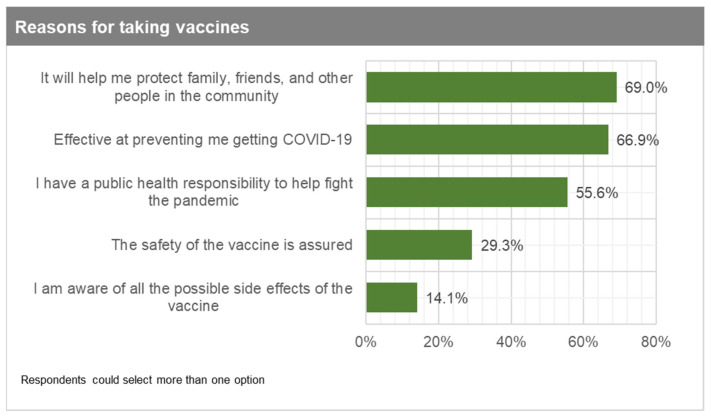
Reasons for taking vaccines.

**Figure 3:**
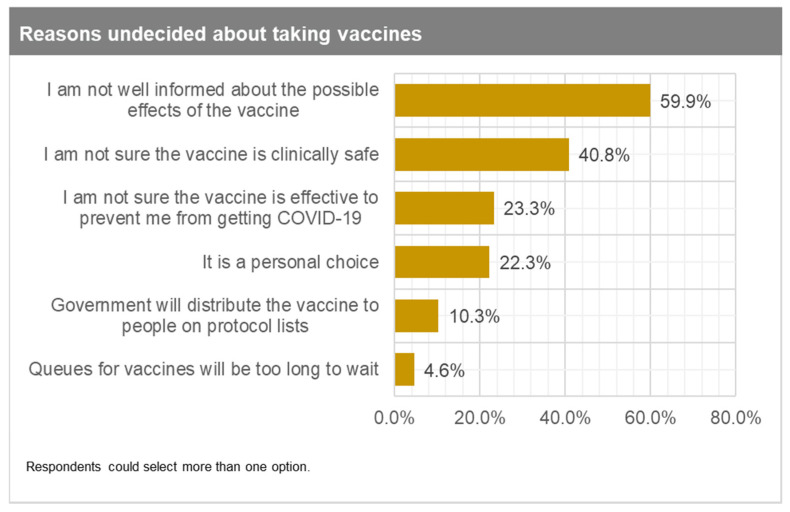
Reasons undecided about taking vaccines.

**Figure 4:**
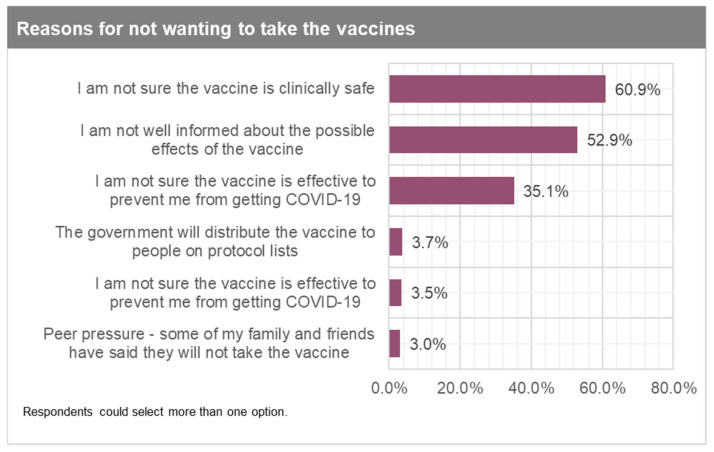
Reasons for not wanting to take the vaccines.

The top three reasons for those very likely or somewhat likely to take the vaccines are: (1) it will help me protect family, friends, and other people in the community (69%); (2) the vaccine is effective at preventing me from getting COVID-19 (67%); and (3) I have a public health responsibility to help fight the pandemic (Figure 2). For those who are undecided, the main reasons they provided were: (1) not being well informed about the possible effects of the vaccine (60%); (2) not being sure that the vaccine is clinically safe (41%); and (3) not being sure that the vaccine is effective to prevent them from getting COVID-19 (23%) (Figure 3). Respondents did not highly rank the option that the government will distribute the vaccine to people on protocol lists, which is very reassuring. Finally, those somewhat unlikely or very unlikely to take the vaccine gave the following top three reasons: (1) I am not sure that the vaccine is clinically safe (61%); (2) I am not well informed about the possible effects of the vaccine (53%); and (3) not being sure that the vaccine is effective to prevent them from getting COVID-19 (35%) (Figure 4). Again, respondents did not highly rank the option of the government distributing vaccines to people on protocol lists.

### 3.3. Factors Associated with COVID-19 Vaccination Intention

To determine the factors that influence the likelihood of Ghanaian adults’ decision to take, remain undecided, or not take the COVID-19 vaccine, we estimated the multinomial logistic regression with the result presented in Table 2. The odds (relative risk) ratios and the marginal effects are reported with their respective 95% confidential interval presented in parenthesis. Two separate models are estimated to highlight the robustness of our modelling approach.

Model I presents the regression output from the weighted sample, while Model II is the regression output from the unweighted sample. Both models include controls for age, educational status, and gender of the respondents as well as region and primary source of information. The original sample (Model II) represents the spatial prevalence of the regional COVID-19 incidence data. This is because Greater Accra (64.48%) and Ashanti (9.34%) comprise a combined 74% of the sample. At the time of completing the data collection on 28 February 2021, these two regions collectively accounted for 73.69% of all of Ghana’s 84,750 total cases. That is, 47,705 cases (56.29%) in Greater Accra and 14,746 cases (17.4%) in Ashanti. However, our results are discussed, emphasising the weighted sample (Model I) as this is the national picture or population sample.

We did not find any statistically significant differences in the region of residence variable on both the weighted and unweighted samples on being undecided or not likely to take the vaccine. This was the same for education. However, the decision to take, remain undecided, or unlikely to take the COVID-19 vaccine is significantly influenced by age, gender, and primary source of information. Relative to respondents aged 15–25 years, Ghanaian adults’ aged 55 years and above are less likely to remain undecided (rrr = 0.466) or unlikely (rrr = 0.473) about their decision to take the vaccine holding other factors constant. The low level of vaccine hesitancy among older age groups can be explained by underlining health conditions associated with old age, thus pushing the aged to be more accepting of vaccines. This aligns with previous studies that found an association between old age and vaccine hesitancy [74].

Regarding gender, we find that the average marginal effect is negative (−0.0694) for the undecided category. This means that the probability of males relative to females remaining undecided on taking the COVID-19 vaccine is on average about 7% lower. When it comes to the unlikely category, again, being male decreases the odds of not taking the vaccine by about two percentage points (−0.0190); however, this is not statistically significant. Previous studies also point out that females have a lower likelihood of deciding to be vaccinated [75,76]. This is because the decision to take a vaccine is considered a risk, and gender-based studies assert that women are more meticulous and will take longer to assess the safety and efficacy of vaccines before being vaccinated [77,78].

Given the role of media and other information sources on information dissemination, it has become incumbent on us to analyse the role of information sources on vaccine hesitancy. Relative to official publications, respondents who obtained information from newspapers are more likely to be undecided (hesitant) on taking the COVID-19 vaccine. Similarly, the study points out that Ghanaian adults who obtained information from newspapers relative to official publications are 0.220 times more unlikely (remain unlikely) to take the vaccine. In line with our expectation, information from official sources such as journals and books would have been fact-checked to ensure high accuracy not to put out anti-vaccine information that will deter people from taking vaccines. It is, therefore, necessary for media houses to vigorously promote the publication of COVID-19 vaccine-based contents from the WHO or the Ghana Health Service for public consumption. Different sources of information also have a significant association with vaccine hesitancy among Ghanaians. Contrary to official sources such as journals and books, information from TV and social media increases the likelihood of not deciding to take the vaccine at varying degrees. Often, information from social media outlets lacks proper scrutiny or scientific vetting and may denote personal opinions, which may be anti-vaccine [79,80].

**Table 2:** Multinomial logistic regression model for intention to accept COVID-19 vaccine.

Explanatory Variable	Model I (Weighted Sample)				Model II (Unweighted Sample)			
	Not Decided		Unlikely		Not Decided		Unlikely	
	Odds Ratio (95% CI)	Average Marginal Effect (95% CI)	Odds Ratio (95% CI)	Average Marginal Effect (95% CI)	Odds Ratio (95% CI)	Average Marginal Effect (95% CI)	Odds Ratio (95% CI)	Average Marginal Effect (95% CI)
**Age**								
Ref: 15–25 years								
26–35 years	0.796 (0.42–1.49)	−0.021 (−0.13–0.09)	0.689 (0.36–1.31)	−0.048 (−0.15–0.05)	0.798 (0.52–1.22)	−0.012 (−0.09–0.09)	0.609 (0.39–0.95) **	−0.073 (−0.15–0.003) *
36–45 years	1.098 (0.56–2.14)	0.015 (−0.11–0.14)	1.051 (0.53–2.10)	0.002 (−0.11–0.11)	0.888 (0.57–1.39)	0.004 (−0.08–0.08)	0.657 (0.41–1.05) *	−0.067 (−0.15–0.01)
46–55 years	1.067 (0.51–2.25)	0.050 (−0.09–0.19)	0.564 (0.25–1.27)	−0.092 (−0.21–0.02)	0.680 (0.41–1.13)	−0.24 (−0.11–0.06)	0.411 (0.24–0.72) ***	−0.12 (−0.21–0.03) ***
>55 years	0.466 (0.23–0.95) **	−0.098 (−0.22–0.02)	0.473 (0.22–0.003) *	−0.079 (−0.19–0.03)	0.528 (0.33–0.74) ***	−0.059 (−0.14–0.02)	0.309 (0.18–0.52) ***	−0.145 (−0.22–0.07) ***
**Education**								
Ref: JSS								
Secondary	0.379 (0.09–1.56)	−0.178 (−0.46–0.10)	0.823 (0.16–4.25)	0.020 (−0.19–0.23)	0.599 (0.21–1.71)	−0.094 (−0.31–0.13)	0.756 (0.21–2.71)	−0.010 (−0.18–0.16)
Tertiary	0.839 (0.25– 2.82)	−0.059 (−0.32–0.20)	1.367 (0.32–5.90)	0.058 (−0.13–0.24)	0.612 (0.23–1.60)	−0.099 (−0.30–0.10)	0.887 (0.28–2.86)	0.013 (−0.15–0.17)
Postgraduate	0.467 (0.14–1.60)	−0.149 (−0.41–0.11)	0.922 (0.21–4.05)	0.028 (−0.16–0.21)	0.380 (0.14–1.00) **	−0.170 (−0.37–0.03)	0.633 (0.20–2.06)	−0.014 (−0.7–0.15)
Other	2.180 (0.28–17.05)	−0.005 (−0.40–0.39)	5.837 (0.62–55.00)	0.251 (−0.11–0.61)	2.001 (0.52–7.72)	0.102 (−0.19–0.39)	2.052 (0.41–10.17)	0.054 (−0.18–0.29)
**Gender**								
Ref: Female								
Male	0.643 (0.47–0.87) ***	−0.069 (−0.13–0.01) **	0.753 (0.53–1.07)	−0.019 (−0.07–0.03)	0.655 (0.53–0.80) ***	−0.065 (−0.10–0.03) ***	0.746 (0.59–0.94) **	−0.022 (−0.06–0.01)
**Region**								
Ref: Ahafo								
Ashanti	0.526 (0.12–2.41)	−0.156 (−0.50–0.19)	1.422 (0.14–14.47)	0.081 (−0.16–0.32)	0.810 (0.15–4.24)	−0.049 (−0.37–0.28)	1.177 (0.12–11.13)	0.034 (−0.26–0.33)
Bono	0.712 (0.13–3.77)	−0.103 (−0.47–0.26)	1.597 (0.14–18.63)	0.081 (−0.19–0.35)	1.311 (0.22–7.90)	0.040 (−0.32–0.40)	1.355 (0.12–14.72)	0.028 (−0.28–0.34)
Bono East	0.548 (0.09–3.46)	−0.168 (−0.55–0.22)	1.982 (0.15–26.86)	0.136 (−0.18–0.45)	0.779 (0.11–5.34)	−0.065 (−0.43–0.30)	1.398 (0.11–17.06)	0.064 (−0.28–0.41)
Central	0.461 (0.09–2.24)	−0.193 (−0.54–0.15)	1.815 (0.17–19.09)	0.130 (−0.12–0.38)	0.733 (0.13–4.03)	−0.079 (−0.41–0.25)	1.483 (0.15–14.45)	0.078 (−0.22–0.38)
Eastern	0.781 (0.17–3.65)	−0.060 (−0.41–0.29)	1.085 (0.10–11.43)	0.022 (−0.22–0.26)	1.181 (0.22–6.31)	0.037 (−0.29–0.37)	0.969 (0.10–9.54)	−0.012 (−0.31–0.28)
GreaterAccra	0.478 (0.11–2.11)	−0.164 (−0.50–0.17)	1.166 (0.12–11.31)	0.056 (−0.18–0.29)	0.742 (0.14–3.78)	−0.056 (−0.38–0.26)	0.969 (0.11–8.93)	0.010 (−0.28–0.30)
North East	0.260 (0.02–4.04)	−0.255 (−0.70–0.19)	1.019 (0.04–25.00)	0.059 (−0.33–0.45)	0.599 (0.04–10.02)	−0.098 (−0.57–0.38)	1.122 (0.05–26.60)	0.041 (−0.41–0.49)
Northern	0.926 (0.19–4.54)	−0.083 (−0.44–0.27)	2.571 (0.23–28.25)	0.145 (−0.12–0.41)	1.316 (0.23–7.43)	0.018 (−0.32–0.36)	1.850 (0.18–18.76)	0.080 (−0.23–0.39)
Oti	0.778 (0.14–4.43)	−0.075 (−0.46–0.31)	1.386 (0.11–17.04)	0.054 (−0.22–0.33)	1.089 (0.17–7.12)	0.007 (−0.36–0.38)	1.205 (0.10–14.52)	0.022 (−0.31–0.35)
Savannah	0.289 (0.03–2.46)	−0.274 (−0.65–0.11)	2.312 (0.16–32.79)	0.203 (−0.15–0.56)	0.446 (0.05–4.18)	−0.154 (−0.52–0.21)	1.610 (0.13–20.24)	0.118 (−0.25–0.49)
Upper East	0.558 (0.10–3.01)	−0.154 (−0.52–0.21)	1.677 (0.15–19.37)	0.105 (−0.16–0.37)	0.902 (0.15–5.44)	−0.036 (−0.38–0.31)	1.345 (0.13–14.20)	0.050 (−0.26–0.36)
Upper West	0.525 (0.10–2.69)	−0.146 (−0.50–0.21)	1.155 (0.10–13.84)	0.050 (−0.22–0.32)	0.729 (0.13–4.25)	−0.052 (−0.39–0.29)	0.812 (0.08–8.77)	−0.013 (−0.32–0.29)
Volta	1.191 (0.24–5.93)	−0.064 (−0.42–0.29)	3.675 (0.35–38.85)	0.192 (−0.07–0.45)	1.986 (0.35–11.19)	0.063 (−0.28–0.40)	3.03 (0.30–30.26)	0.137 (−0.17–0.44)
Western	0.540 (0.11–2.64)	−0.156 (−0.51–0.19)	1.565 (0.15–16.61)	0.095 (−0.16–0.35)	0.875 (0.16–4.84)	−0.040 (−0.37–0.29)	1.292 (0.13–12.79)	0.045 (−0.26–0.35)
Western North	0.876 (0.13–5.95)	−0.043 (−0.46–0.38)	1.268 (0.07–22.09)	0.035 (−0.28–0.35)	1.22 (0.17–901)	0.051 (−0.35–0.45)	0.842 (0.05–13.11)	−0.031 (−0.37–0.30)
**Primary Info Source**								
Ref: Official Publications								
Media-TV	1.085 (0.56–2.09)	−0.018 (−0.14–0.11)	1.99 (0.89–4.44) *	0.087 (0.003–0.17) **	1.102 (0.72–1.69)	0.019 (−0.05–0.09)	0.981 (0.60–1.59)	−0.007 (−0.08–0.06)
Newspapers	0.453 (0.14–1.48)	−0.178 (−0.34–0.02) **	3.105 (1.05–9.19) **	0.220 (0.05–0.39) **	0.592 (0.26–1.34)	−0.087 (−0.20–0.02)	1.190 (0.58–2.45)	0.050 (−0.06–0.16)
Social media	0.978 (0.49–1.93)	−0.034 (−0.16–0.10)	1.848 (0.81–4.20)	0.081 (−0.006–0.17) *	1.188 (0.76–1.87)	0.028 (−0.05–0.11)	1.082 (0.65–1.80)	0.003 (−0.07–0.08)
Word of mouth	1.080 (0.36–3.28)	−0.026 (−0.23–0.18)	2.218 (0.72–6.87)	0.105 (−0.05–0.26)	0.930 (0.44–1.97)	−0.023 (−0.15–0.10)	1.257 (0.57–2.76)	0.040 (−0.08–0.16)

Significance thresholds: *p* < 1 *, *p* < 0.05 **, *p* < 0.01 ***.

## 4. Discussion

In general, vaccine acceptance levels are about half of the sampled adult population. Our survey results suggest that chances of attaining the coveted 63–70% herd immunity threshold in Ghana would be enhanced if the preventive vaccination programmes, which are underway in-country, are combined with an enhanced and coordinated public education campaign so that at least 19.57 million Ghanaians and residents are vaccinated (or achieve immunity) within an acceptable time frame (Table 3). However, using our 51% of the adult population over 15 years who are likely to take the COVID-19 vaccine means that only 9.98 million persons (19.57 million * 51%) are likely to take the vaccine (Table 3). This means that potentially 9.59 million persons or 49% of the 19.57 million population over 15-years needed to attain herd immunity are undecided or will likely not take the vaccine—which must be a concern. Using the 70% total population as the basis for herd immunity increases the number to 11.77 million potentially undecided or unlikely citizens who will not take the vaccine. It is also further complicated because there are no vaccines licensed as yet in Ghana for people below 16-years.

One important consideration we have not factored so far in our analysis is the seroprevalence or presence of COVID-19 antibodies accounted for by naturally acquired immunity. Several studies have reported detectable SARS-CoV-2 antibodies (seropositivity) of up to 25% [81,82,83]. In sub-Saharan Africa, a 25.41% seroprevalence rate was estimated in Niger State, Nigeria [84]. Also, in Zambia, a 10.6% SARS-CoV-2 prevalence based on sampling in six districts in Zambia in July 2020 is reported [85]. In Kenya, there is a reported a 4.3% population-weighted and test performance-adjusted national seroprevalence rate [86]. Additionally, George et al. [87] reported 27.8% mean age and test adjusted SARS CoV2 seroprevalence in the Gauteng province in South Africa. Given the foregoing, we simulated two scenarios of how naturally acquired immunity will influence vaccine acquisition and rollout (Table 3). Assuming 10% naturally acquired immunity, 8.40 million of Ghana’s adult population more than 15 years are likely to take the vaccine, while another 8.07 million are unlikely to take the vaccine. Assuming 20% naturally acquired immunity, 6.81 million of Ghana’s adult population over 15 years are likely to take the vaccine, while another 6.55 million are unlikely to take the vaccine. Thus, factoring in naturally acquired immunity means slightly lower vaccines would need to be procured.

Despite the preceding estimates, the inclusion of the “undecided” and “somewhat likely” adult population will significantly improve vaccine uptake and herd immunity dynamics. Hence, the next best thing will be for all the eligible 63% of the adult population above 15 years to be vaccinated. Public campaigns should involve multiple channels but strongly emphasise media (TV and radio) and social media (Facebook, WhatsApp, Twitter, among others). These are the primary sources for acquiring information or knowledge among our respondents. Additionally, nuanced messaging emphasising the efficacy and safety of the vaccine should be strongly considered. These are the main reasons people are undecided or unwilling to take the vaccine, as shown earlier in Figure 3 and Figure 4. Such messaging should also consider the demographic differences in Ghana’s population.

Our study has some limitations. First, the study has a higher proportion of respondents in the 26–35 years and 36–45 year group than in the general population. This is because there was a mismatch of one additional year between Ghana’s regional population group breakdown as provided in official statistics and the age categorisation we used. For example, while we use 26–35 and 36–45 years for age categorisation, Ghana’s official data has 25–34 years, 35–44 years and other similar categories. So, the iterative proportional fitting was specified using only the regional population totals and regional gender split. The algorithm subsequently adjusted this for age and education, albeit resulting in imperfect representation. Other than the age group distribution, the demographics of the respondents were comparable with Ghana’s population and largely urbanised structure.

Secondly, while the data [88] shows that there are about 16 million internet users in Ghana with about 50% internet penetration and over 100% mobile phone penetration, the lack of efficient internet communication means a segment of the rural population may have been excluded from the survey. Additionally, generally lower levels of education in Ghana’s rural areas would likely lead to less vaccination acceptance in these areas—for example, as demonstrated in [89,90]. Thirdly, this being a cross-sectional survey means that it is wholly impossible to determine causal relationships between the factors and dependent variables. Future research could consider a longitudinal cohort study with additional time points to ascertain time-varying willingness to vaccinate. This would allow policymakers to see how the changing picture of prevalence may alter vaccine hesitancy. Fourthly, our questions focused on vaccination provided by the government. However, responses have been different based on sources such as the private sector or companies providing them directly to their employees. Finally, we did not ask questions related to vaccine hesitancy based on the type of vaccines, such as between mRNA vaccines (such as Pfizer-BionTech, Moderna) and adenovirus vector vaccines (such as AstraZeneca-University of Oxford, Johnson & Johnson, Sputnik V), for example. Despite these limitations, the reported vaccine acceptance rates were similar to other reported studies, as noted in earlier parts of this paper.

## 5. Conclusions

This study examined vaccine hesitancy attitudes among mostly urban adult Ghanaians and explored the likelihood of participation or non-participation in the government’s effort to vaccinate citizens. We find that about 5 in 10 (51%) of mostly urban Ghanaians over 15 years are likely to take the COVID-19 vaccine if it was made generally available. We also found that almost a fifth (21%) of the respondents were unlikely to take the vaccine, while another 28% were undecided. Additionally, we find differences in vaccine hesitancy and some socio-demographic characteristics. For example, there are low vaccine hesitancy levels among older age groups, while males are more likely to be undecided about taking the vaccine than females. No significant associations were found between willingness to vaccinate and education or region of residence. Attaining the coveted 63–70% herd immunity threshold in Ghana would be enhanced if the preventive vaccination programmes, which are underway in-country, are combined with an enhanced and coordinated public education campaign. Thus, there is an urgent need for key stakeholders in the health sector to increase efforts in targeted public education and raise awareness about the individual and collective benefit of vaccinations, especially among younger populations, with an additional focus on men. Such a campaign should focus on curbing misinformation about vaccines.

## Figures and Tables

**Table 1:** Participant socio-demographics and the likelihood of taking COVID-19 vaccine.

Variable	Total Weighted Sample N (%)	Likelihood of Taking COVID-19 Vaccine			
		Likely	Undecided	Unlikely	*p*-Value
Total sample	2345 (100)	1197 (51%)	662 (28%)	486 (21%)	-
**Gender**					
Female	1122 (48%)	518 (46%)	359 (32%)	245 (22%)	0.0083 ***
Male	1223 (52%)	679 (56%)	303 (25%)	241 (20%)	
**Age group**					
15–25	177 (8%)	70 (40%)	59 (34%)	47 (27%)	0.0068 ***
26–35	1104 (47%)	565 (51%)	311 (28%)	228 (21%)	
36–45	546 (23%)	251 (46%)	163 (30%)	132 (24%)	
46–55	208 (9%)	107 (51%)	69 (33%)	32 (16%)	
Above 55	310 (13%)	203 (66%)	60 (19%)	47 (15%)	
**Education**					
Junior Secondary (JSS)	30 (1%)	11 (38%)	13 (44%)	5 (18%)	0.0042 ***
Senior Secondary (SSS)	103 (4%)	64 (62%)	21 (21%)	18 (18%)	
Tertiary	1339 (57%)	619 (46%)	425 (32%)	295 (22%)	
Postgraduate	852 (36%)	499 (59%)	194 (23%)	159 (19%)	
Other	21 (1%)	4 (21%)	8 (37%)	9 (41%)	
**Tested for COVID-19**					
No	1729 (74%)	826 (48%)	524 (30%)	379 (22%)	0.0013 ***
Yes	615 (26%)	371 (60%)	138 (22%)	107 (17%)	
**Primary Source of Information**					
Books/Journals/Official Publications	124 (5%)	73 (59%)	35 (28%)	16 (13%)	0.3970
Media-TV	1364 (58%)	692 (51%)	391 (29%)	281 (20%)	
Newspapers/Print Media	74 (3%)	38 (52%)	10 (13%)	25 (35%)	
Social Media	708 (30%)	358 (51%)	203 (29%)	147 (21%)	
Word of Mouth (Family and Friends)	76 (3%)	36 (48%)	23 (31%)	17 (22%)	
**Region**					
Ahafo	48 (2%)	22 (45%)	20 (42%)	6 (12%)	0.7493
Ashanti	452 (19%)	247 (55%)	113 (25%)	92 (20%)	
Bono	91 (4%)	43 (47%)	30 (33%)	18 (20%)	
Bono East	79 (3%)	37 (47%)	21 (26%)	21 (26%)	
Central	200 (9%)	101 (51%)	47 (24%)	52 (26%)	
Eastern	251 (11%)	127 (51%)	89 (35%)	35 (14%)	
Greater Accra	464 (20%)	268 (58%)	116 (25%)	80 (17%)	
North East	35 (1%)	23 (65%)	6 (17%)	6 (17%)	
Northern	125 (5%)	51 (41%)	43 (34%)	31 (25%)	
Oti	51 (2%)	24 (47%)	18 (35%)	9 (18%)	
Savannah	34 (1%)	18 (55%)	4 (13%)	11 (32%)	
Upper East	85 (4%)	43 (50%)	22 (26%)	20 (24%)	
Upper West	49 (2%)	26 (53%)	14 (27%)	10 (19%)	
Volta	151 (6%)	49 (33%)	54 (36%)	48 (32%)	
Western	162 (7%)	84 (52%)	41 (25%)	37 (23%)	
Western North	69 (3%)	33 (48%)	25 (37%)	11 (16%)	

Significance thresholds: *p* < 1 *, *p* < 0.05 **, *p* < 0.01 ***.

**Table 3:** Ghana’s herd immunity simulation.

Variable	Assuming No Naturally Acquired Immunity		Assuming 10% Naturally Acquired Immunity		Assuming 20% Naturally Acquired Immunity	
	Population (Millions)	Percentage (%)	Population (Millions)	Percentage (%)	Population (millions)	Percentage (%)
**(A) Ghana 2020 total population (GSS)**	31.07	100.00%	31.07	100.00%	31.07	100.00%
**(B) % of the total population needed for herd immunity**	21.75	70.00%	18.64	60.00%	15.54	50.00%
**(C) Ghana’s adult population more than 15 years (GSS)**	19.57	63.00%	16.47	53.00%	13.36	43.00%
**(D) Adult population more than 15 years likely to take vaccine (from survey)**	9.98	51.00%	8.40	51.00%	6.81	51.00%
**(E) Difference (C–D)**	9.59	49.00%	8.07	49.00%	6.55	49.00%

## Data Availability

We have made the data presented in this study openly available under CC BY 4.0 license on the Mendeley platform at doi: 10.17632/mzpt4ytj7m.1.

## References

[1] WHO (2020). WHO Director-General’s Opening Remarks at the Media Briefing on COVID-19—11 March 2020. https://www.who.int/director-general/speeches/detail/who-director-general-s-opening-remarks-at-the-media-briefing-on-covid-19---11-march-2020.

[2] Ghana Health Service (2021). COVID-19 Updates. https://www.ghanahealthservice.org/covid19/archive.php.

[3] Colbourn T. (2020). COVID-19: Extending or relaxing distancing control measures. Lancet Public Health.

[4] Hartley D.M., Perencevich E.N. (2020). Public health interventions for COVID-19: Emerging evidence and implications for an evolving public health crisis. JAMA.

[5] Pan A., Liu L., Wang C., Guo H., Hao X., Wang Q., Huang J., He N., Yu H., Lin X. (2020). Association of public health interventions with the epidemiology of the COVID-19 outbreak in Wuhan, China. JAMA.

[6] Fisher D., Teo Y.Y., Nabarro D. (2020). Assessing national performance in response to COVID-19. Lancet.

[7] Scally G., Jacobson B., Abbasi K. (2020). The UK’s public health response to covid-19. BMJ.

[8] Schuchat A., CDC COVID-19 Response Team (2020). Public health response to the initiation and spread of pandemic COVID-19 in the United States, 24 February–21 April, 2020. Morb. Mortal. Wkly. Rep..

[9] Tabari P., Amini M., Moghadami M., Moosavi M. (2020). International public health responses to COVID-19 outbreak: A rapid review. Iran. J. Med Sci..

[10] Thayer W.M., Hasan M.Z., Sankhla P., Gupta S. (2021). An interrupted time series analysis of the lockdown policies in India: A national-level analysis of COVID-19 incidence. Health Policy Plan..

[11] Frempong N.K., Acheampong T., Apenteng O.O., Nakua E., Amuasi J.H. (2021). Does the data tell the true story? A modelling study of early COVID-19 pandemic suppression and mitigation strategies in Ghana. medRxiv.

[12] Quakyi N.K. (2021). Ghana’s COVID-19 Vaccine Rollout Is Struggling to Keep Up with Its Great Start. https://theconversation.com/ghanas-covid-19-vaccine-rollout-is-struggling-to-keep-up-with-its-great-start-159579.

[13] Quakyi N.K., Asante N.A.A., Nartey Y.A., Bediako Y., Sam-Agudu N.A. (2021). Ghana’s COVID-19 response: The Black Star can do even better. BMJ Glob. Health.

[14] Bloom D.E. (2011). The value of vaccination. Hot Topics in Infection and Immunity in Children VII.

[15] Londono S.E., Li X., Toor J., de Villiers M.J., Nayagam S., Hallett T.B., Gaythorpe K.A. (2021). How can the public health impact of vaccination be estimated?. medRxiv.

[16] Preaud E., Durand L., Macabeo B., Farkas N., Sloesen B., Palache A., Samson S.I. (2014). Annual public health and economic benefits of seasonal influenza vaccination: A European estimate. BMC Public Health.

[17] Randolph H.E., Barreiro L.B. (2020). Herd immunity: Understanding COVID-19. Immunity.

[18] WHO (2020). Coronavirus Disease (COVID-19): Herd Immunity, Lockdowns and COVID-19. https://www.who.int/news-room/q-a-detail/herd-immunity-lockdowns-and-covid-19.

[19] D’souza G., Dowdy D. (2021). What Is Herd Immunity and How Can We Achieve It With COVID-19? Johns Hopkins Bloomberg School of Public Health. https://www.jhsph.edu/covid-19/articles/achieving-herd-immunity-with-covid19.html.

[20] Aschwanden C. (2021). Five reasons why COVID herd immunity is probably impossible. Nature.

[21] McNeil D.G. (2020). How Much Herd Immunity Is Enough?. https://www.nytimes.com/2020/12/24/health/herd-immunity-covid-coronavirus.html.

[22] WHO (2020). Episode #1—Herd immunity. https://www.who.int/emergencies/diseases/novel-coronavirus-2019/media-resources/science-in-5/episode-1.

[23] MacDonald N.E. (2015). Vaccine hesitancy: Definition, scope and determinants. Vaccine.

[24] Dubé E., MacDonald N.E. (2020). How can a global pandemic affect vaccine hesitancy?. Expert Rev. Vaccines.

[25] Dubé E., Laberge C., Guay M., Bramadat P., Roy R., Bettinger J.A. (2013). Vaccine hesitancy: An overview. Hum. Vaccines Immunother..

[26] Dubé E., Vivion M., MacDonald N.E. (2015). Vaccine hesitancy, vaccine refusal and the anti-vaccine movement: Influence, impact and implications. Expert Rev. Vaccines.

[27] MacDonald N.E., Butler R., Dubé E. (2018). Addressing barriers to vaccine acceptance: An overview. Hum. Vaccines Immunother..

[28] Yaqub O., Castle-Clarke S., Sevdalis N., Chataway J. (2014). Attitudes to vaccination: A critical review. Soc. Sci. Med..

[29] Jennings W., Stoker G., Bunting H., Valgarðsson V.O., Gaskell J., Devine D., McKay L., Mills M.C. (2021). Lack of trust, conspiracy beliefs, and social media use predict COVID-19 vaccine hesitancy. Vaccines.

[30] Murphy J., Vallières F., Bentall R.P., Shevlin M., McBride O., Hartman T.K., McKay R., Bennett K., Mason L., Gibson-Miller J. (2021). Psychological characteristics associated with COVID-19 vaccine hesitancy and resistance in Ireland and the United Kingdom. Nat. Commun..

[31] Peretti-Watel P., Seror V., Cortaredona S., Launay O., Raude J., Verger P., Fressard L., Beck F., Legleye S., L’Haridon O. (2020). A future vaccination campaign against COVID-19 at risk of vaccine hesitancy and politicisation. Lancet Infect. Dis..

[32] Sallam M. (2021). COVID-19 vaccine hesitancy worldwide: A concise systematic review of vaccine acceptance rates. Vaccines.

[33] Troiano G., Nardi A. (2021). Vaccine hesitancy in the era of COVID-19. Public Health.

[34] Cerda A.A., García L.Y. (2021). Hesitation and refusal factors in individuals’ decision-making processes regarding a Coronavirus disease 2019 vaccination. Front. Public Health.

[35] Dror A.A., Eisenbach N., Taiber S., Morozov N.G., Mizrachi M., Zigron A., Srouji S., Sela E. (2020). Vaccine hesitancy: The next challenge in the fight against COVID-19. Eur. J. Epidemiol..

[36] WHO (2014). Report of the Sage Working Group on Vaccine Hesitancy. https://www.who.int/immunization/sage/meetings/2014/october/1_Report_WORKING_GROUP_vaccine_hesitancy_final.pdf.

[37] Wiysonge C.S., Ndwandwe D., Ryan J., Jaca A., Batouré O., Anya B.P.M., Cooper S. (2021). Vaccine hesitancy in the era of COVID-19: Could lessons from the past help in divining the future?. Hum. Vaccines Immunother..

[38] Khubchandani J., Sharma S., Price J.H., Wiblishauser M.J., Sharma M., Webb F.J. (2021). COVID-19 vaccination hesitancy in the United States: A rapid national assessment. J. Community Health.

[39] Chen M., Li Y., Chen J., Wen Z., Feng F., Zou H., Fu C., Chen L., Shu Y., Sun C. (2021). An online survey of the attitude and willingness of Chinese adults to receive COVID-19 vaccination. Hum. Vaccines Immunother..

[40] Dzinamarira T., Nachipo B., Phiri B., Musuka G. (2021). COVID-19 vaccine roll-out in South Africa and Zimbabwe: Urgent need to address community preparedness, fears and hesitancy. Vaccines.

[41] Gagneux-Brunon A., Detoc M., Bruel S., Tardy B., Rozaire O., Frappe P., Botelho-Nevers E. (2021). Intention to get vaccinations against COVID-19 in French healthcare workers during the first pandemic wave: A cross-sectional survey. J. Hosp. Infect..

[42] Agyekum M.W., Afrifa-Anane G.F., Kyei-Arthur F., Addo B. (2021). Acceptability of COVID-19 vaccination among health care workers in Ghana. Adv. Public Health.

[43] Reuters (2021). Ghana Aims to Get 17.6 Million Doses of COVID-19 Vaccine by June. https://www.reuters.com/article/uk-health-coronavirus-ghana-idUSKBN2A11KB.

[44] ModernGhana (2021). Ghana on Course to Procure 42 Million More COVID-19 vaccines—Akufo-Addo Assures. https://www.modernghana.com/news/1070581/ghana-on-course-to-procure-42-million-more-covid.html.

[45] Sevencan S. (2021). Ghana to Receive over 1M Doses of Sputnik V Vaccine. https://www.aa.com.tr/en/africa/ghana-to-receive-over-1m-doses-of-sputnik-v-vaccine/2227628.

[46] BBC (2021). Covax Vaccine-Sharing Scheme Delivers First Doses to Ghana. https://www.bbc.co.uk/news/world-africa-56180161.

[47] WHO (2021). COVID-19 Vaccine Doses Shipped by the COVAX Facility Head to Ghana, Marking Beginning of Global Rollout. https://www.who.int/news/item/24-02-2021-covid-19-vaccine-doses-shipped-by-the-covax-facility-head-to-ghana-marking-beginning-of-global-rollout.

[48] Dontoh E. (2021). Ghana Gets Second Batch of AstraZeneca Vaccines from Covax. https://www.bloomberg.com/news/articles/2021-05-07/ghana-gets-second-batch-of-astrazeneca-vaccines-from-covax.

[49] WHO (2021). Emerging Lessons from Africa’s COVID-19 Vaccine Rollout—Ghana. https://www.afro.who.int/news/emerging-lessons-africas-covid-19-vaccine-rollout.

[50] Afolabi A.A., Ilesanmi O.S. (2021). Dealing with vaccine hesitancy in Africa: The prospective COVID-19 vaccine context. Pan. Afr. Med. J..

[51] Rufai N.A. (2021). After Botched Ebola Vaccine Trial, Ghana Struggles with Vaccine Hesitancy. https://www.pbs.org/newshour/show/after-botched-ebola-vaccine-trial-ghana-struggles-with-vaccine-hesitancy.

[52] Johnson T.P. (2014). Snowball Sampling: Introduction. Wiley StatsRef: Statistics Reference Online. https://onlinelibrary.wiley.com/doi/abs/10.1002/9781118445112.stat05720.

[53] Carrieri V., Madio L., Principe F. (2019). Vaccine hesitancy and (fake) news: Quasi-experimental evidence from Italy. Health Econ..

[54] Hansen P.R., Schmidtblaicher M. (2021). A dynamic model of vaccine compliance: How fake news undermined the Danish HPV vaccine program. J. Bus. Econ. Stat..

[55] Salathé M., Bonhoeffer S. (2008). The effect of opinion clustering on disease outbreaks. J. R. Soc. Interface.

[56] Allcott H., Gentzkow M., Yu C. (2019). Trends in the diffusion of misinformation on social media. Res. Politics.

[57] Apuke O.D., Omar B. (2021). Fake news and COVID-19: Modelling the predictors of fake news sharing among social media users. Telemat. Inform..

[58] Chou W.Y.S., Oh A., Klein W.M. (2018). Addressing health-related misinformation on social media. JAMA.

[59] Rampersad G., Althiyabi T. (2020). Fake news: Acceptance by demographics and culture on social media. J. Inf. Technol. Politics.

[60] World Bank (2021). GNI per Capita, Atlas Method (Current US$)—Ghana | Data. https://data.worldbank.org/indicator/NY.GNP.PCAP.CD?locations=GH.

[61] Ghana Statistical Service (2020). Projected Population by Age and Sex, 260 Districts 2020. https://statsghana.gov.gh/nationalaccount_macros.php?Stats=MTA1NTY1NjgxLjUwNg==/webstats/s679n2sn87.

[62] Abdelhafiz A.S., Mohammed Z., Ibrahim M.E., Ziady H.H., Alorabi M., Ayyad M., Sultan E.A. (2020). Knowledge, perceptions, and attitude of Egyptians towards the novel coronavirus disease (COVID-19). J. Community Health.

[63] Chow M.Y.K., Danchin M., Willaby H.W., Pemberton S., Leask J. (2017). Parental attitudes, beliefs, behaviours and concerns towards childhood vaccinations in Australia: A national online survey. Aust. Fam. Physician.

[64] Larson H.J., Jarrett C., Schulz W.S., Chaudhuri M., Zhou Y., Dube E., Schuster M., MacDonald N.E., Wilson R. (2015). Measuring vaccine hesitancy: The development of a survey tool. Vaccine.

[65] Schwarzinger M., Watson V., Arwidson P., Alla F., Luchini S. (2021). COVID-19 vaccine hesitancy in a representative working-age population in France: A survey experiment based on vaccine characteristics. Lancet Public Health.

[66] Ahmad H., Halim H. (2017). Determining sample size for research activities. Selangor Bus. Rev..

[67] Kasiulevičius V., Šapoka V., Filipavičiūtė R. (2006). Sample size calculation in epidemiological studies. Gerontologija.

[68] Woolson R.F., Bean J.A., Rojas P.B. (1986). Sample size for case-control studies using Cochran’s statistic. Biometrics.

[69] Kolenikov S. (2014). Calibrating survey data using iterative proportional fitting (raking). Stata J..

[70] Mercer A., Lau A., Kennedy C. (2018). How Different Weighting Methods Work—Pew Research Center Method. https://www.pewresearch.org/methods/2018/01/26/how-different-weighting-methods-work.

[71] Greene W.H. (2003). Econometric Analysis.

[72] CDC (2020). COVID-19 and Your Health. https://www.cdc.gov/coronavirus/2019-ncov/need-extra-precautions/older-adults.html.

[73] UK Government (2021). Chapter 14a—COVID-19-SARS-CoV-2. https://assets.publishing.service.gov.uk/government/uploads/system/uploads/attachment_data/file/978508/Green_book_chapter_16April2021.pdf.

[74] Edwards B., Biddle N., Gray M., Sollis K. (2021). COVID-19 vaccine hesitancy and resistance: Correlates in a nationally representative longitudinal survey of the Australian population. PLoS ONE.

[75] Latkin C.A., Dayton L., Yi G., Colon B., Kong X. (2021). Mask usage, social distancing, racial, and gender correlates of COVID-19 vaccine intentions among adults in the US. PLoS ONE.

[76] Latkin C.A., Dayton L., Yi G., Konstantopoulos A., Boodram B. (2021). Trust in a COVID-19 vaccine in the US: A social-ecological perspective. Soc. Sci. Med..

[77] Cordina M., Lauri M.A. (2021). Attitudes towards COVID-19 vaccination, vaccine hesitancy and intention to take the vaccine. Pharm. Pract..

[78] Inglehart R., Norris P. (2000). The developmental theory of the gender gap: Women’s and men’s voting behavior in global perspective. Int. Political Sci. Rev..

[79] Meleo-Erwin Z., Basch C., MacLean S.A., Scheibner C., Cadorett V. (2017). “To each his own”: Discussions of vaccine decision-making in top parenting blogs. Hum. Vaccines Immunother..

[80] Puri N., Coomes E.A., Haghbayan H., Gunaratne K. (2020). Social media and vaccine hesitancy: New updates for the era of COVID-19 and globalised infectious diseases. Hum. Vaccines Immunother..

[81] He Z., Ren L., Yang J., Guo L., Feng L., Ma C., Wang X., Leng Z., Tong X., Zhou W. (2021). Seroprevalence and humoral immune durability of anti-SARS-CoV-2 antibodies in Wuhan, China: A longitudinal, population-level, cross-sectional study. Lancet.

[82] Poustchi H., Darvishian M., Mohammadi Z., Shayanrad A., Delavari A., Bahadorimonfared A., Eslami S., Javanmard S.H., Shakiba E., Somi M.H. (2021). SARS-CoV-2 antibody seroprevalence in the general population and high-risk occupational groups across 18 cities in Iran: A population-based cross-sectional study. Lancet Infect. Dis..

[83] Shakiba M., Nazari S.S.H., Mehrabian F., Rezvani S.M., Ghasempour Z., Heidarzadeh A. (2020). Seroprevalence of COVID-19 virus infection in Guilan province, Iran. medRxiv.

[84] Majiya H., Aliyu-Paiko M., Balogu V.T., Musa D.A., Salihu I.M., Kawu A.A., Bashir I.Y., Sani A.R., Baba J., Muhammad A.T. (2020). Seroprevalence of SARS-CoV-2 in Niger State: A Pilot Cross Sectional Study. Medrxiv.

[85] Mulenga L.B., Hines J.Z., Fwoloshi S., Chirwa L., Siwingwa M., Yingst S., Wolkon A., Barradas D.T., Favaloro J., Zulu J.E. (2021). Prevalence of SARS-CoV-2 in six districts in Zambia in July, 2020: A cross-sectional cluster sample survey. Lancet Glob. Health.

[86] Uyoga S., Adetifa I.M., Karanja H.K., Nyagwange J., Tuju J., Wanjiku P., Aman R., Mwangangi M., Amoth P., Kasera K. (2021). Seroprevalence of anti–SARS-CoV-2 IgG antibodies in Kenyan blood donors. Science.

[87] George J.A., Khoza S., Mayne E., Dlamini S., Kone N., Jassat W., Chetty K., Centner C., Pillay T., Maphayi M.R. (2021). Sentinel seroprevalence of SARS-CoV-2 in the Gauteng province, South Africa August to October 2020. medRxiv.

[88] Kemp S. (2021). Digital in Ghana: All the Statistics You Need in 2021—DataReportal—Global Digital Insights. https://datareportal.com/reports/digital-2021-ghana.

[89] Abedin M., Islam M.A., Rahman F.N., Reza H.M., Hossain M.Z., Hossain M.A., Hossain A. (2021). Willingness to vaccinate against COVID-19 among Bangladeshi adults: Understanding the strategies to optimize vaccination coverage. PLoS ONE.

[90] Le X.T., Nguyen H.T., Le H.T., Do T.T., Nguyen T.H., Vu L.G., Ho R.C. (2021). Rural–urban differences in preferences for influenza vaccination among women of childbearing age: Implications for local vaccination service implementation in Vietnam. Trop. Med. Int. Health.

